# Influence of impaired glucose tolerance alone and combined with metabolic syndrome on long‐term risk of cardiovascular events and mortality

**DOI:** 10.1111/1753-0407.13598

**Published:** 2024-08-19

**Authors:** Fei Chen, Yifan He, Jinping Wang, Liping Yu, Qiuhong Gong, Yanyan Chen, Yali An, Siyao He, Guangwei Li, Bo Zhang

**Affiliations:** ^1^ Department of Endocrinology China‐Japan Friendship Hospital Beijing China; ^2^ Department of Cardiology Da Qing First Hospital Da Qing China; ^3^ Endocrinology Centre Fuwai Hospital, Chinese Academy of Medical Sciences and Peking Union Medical College Beijing China

**Keywords:** cardiovascular events, impaired glucose tolerance, metabolic syndrome, mortality

## Abstract

**Background:**

This study aimed to investigate the potential differences in the influence of impaired glucose tolerance (IGT) with and without metabolic syndrome (MetS) on cardiovascular (CV) events and mortality.

**Methods:**

Participants having IGT with MetS (IGT_MetS), those having IGT without MetS (IGT_non_MetS), and those having normal glucose tolerance (NGT) without MetS (NGT_non_MetS) (*N* = 246, *N* = 294, and *N* = 471, respectively) were included in this study. Cox proportional hazards regression was used to examine the relationship among these three groups and CV events and mortality.

**Results:**

Over the 30‐year follow‐up period, 57 (12.1%) participants having NGT_non_MetS, 55 (18.71%) with IGT_non_MetS, and 74 (30.08%) with IGT_MetS experienced CV mortality. After adjusting for risk factors, the hazard ratios for CV mortality were 2 (95% confidence interval [CI], 1.38–2.91) for the IGT_non_MetS group and 2.96 (95% CI, 2.09–4.19) for the IGT_MetS group, compared with the NGT_non_MetS group. Similar patterns were observed for CV events, with hazard ratios of 1.49 (95% CI, 1.19–1.88) for the IGT_non_MetS group and 1.97 (95% CI, 1.58–2.47) for the IGT_MetS group. Sensitivity analysis revealed that the hazard ratios of the IGT_non_MetS and IGT_MetS groups indicated a higher risk of all‐cause mortality, myocardial infarction events or myocardial infarction mortality, and stroke events or stroke mortality compared with that of the NGT_non_MetS group.

**Conclusion:**

IGT_non_MetS increased the risk of CV mortality and events. Furthermore, when it occurred in conjunction with MetS, it further increased the risk of CV mortality and events. This suggested that active intervention is required.

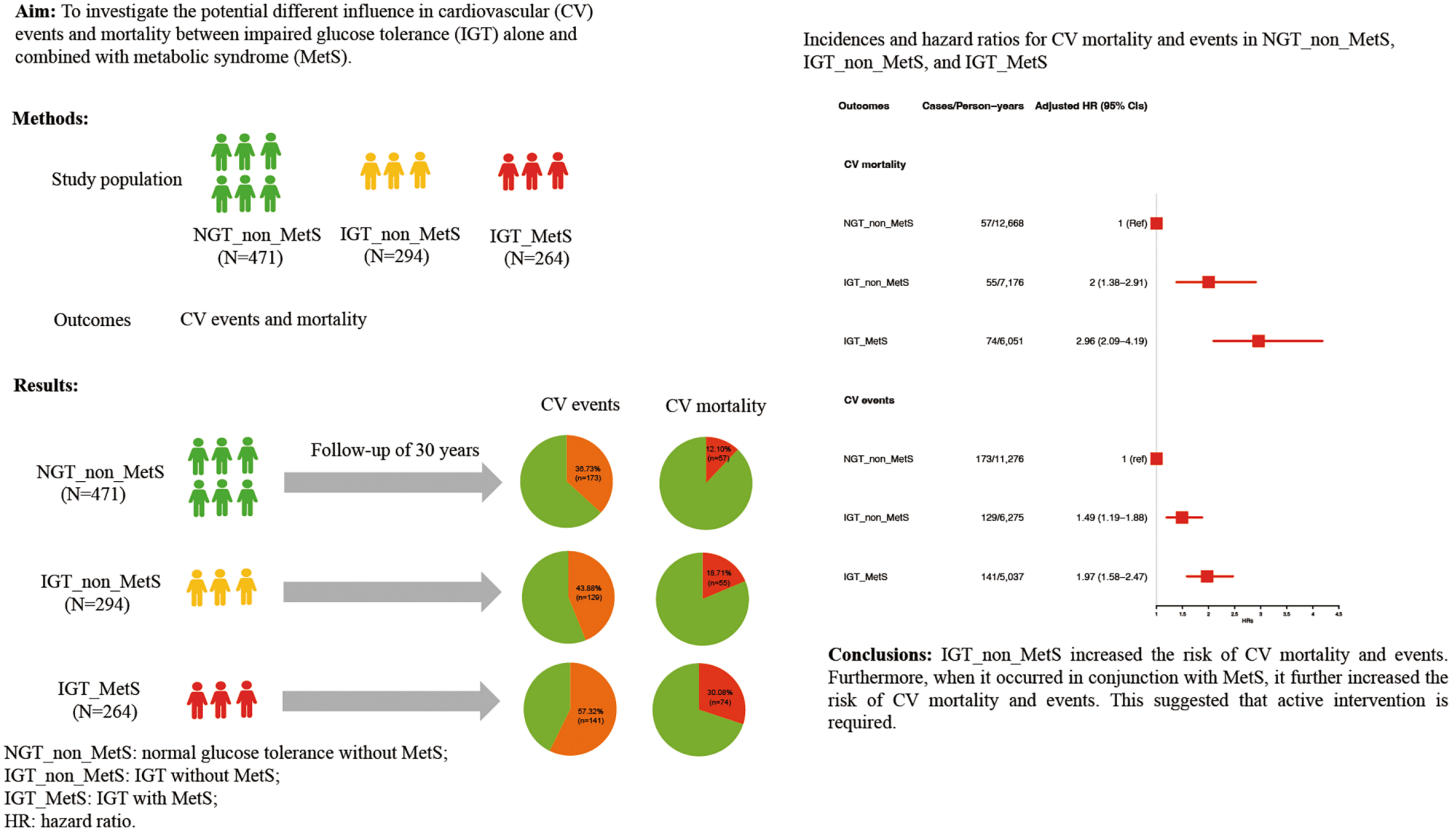

## Graphical abstract

- The IGT_non_MetS or IGT_MetS increased the risk of CV mortality and events.
- We found that the IGT_non_MetS and IGT_MetS groups had higher risk of all‐cause mortality, MI events or MI mortality, and stroke events or stroke mortality compared with the NGT_non_MetS group.
- Tailoring interventions according to whether IGT was accompanied by MetS can potentially maximize cost‐effectiveness, especially within the constraints of limited medical resources.

## 1 INTRODUCTION

Impaired glucose tolerance (IGT) refers to an intermediate metabolic state between normal glucose tolerance (NGT) and diabetes mellitus (DM). Its prevalence in mainland China has been steadily increasing, reaching an estimated rate of 13.4% in 2021.^1^ This highlights the significant burden of IGT in these populations, with individuals in this intermediate state at a heightened risk of developing diabetes.^2^, ^3^ Furthermore, individuals with diabetes are at an increased risk of developing cardiovascular (CV) events and have higher mortality rates compared with those with NGT.^4^, ^5^, ^6^ Notably, emerging evidence suggests that IGT may contribute to an elevated risk of CV events and mortality.^7^

Metabolic syndrome (MetS), a cluster of metabolic abnormalities, including central obesity, dyslipidemia, hypertension, and insulin resistance, is a significant contributor to CV risk.^8^, ^9^ Large meta‐analyses have demonstrated that individuals with MetS have approximately twice the risk of developing CV events compared with those without MetS.^10^, ^11^, ^12^ Given that a considerable proportion of individuals with IGT also have MetS, it is plausible that the association between IGT and CV events and mortality is possibly mediated by the presence of MetS.^13^ Thus, IGT without MetS (IGT_non_MetS) may not be associated with CV events and mortality. However, studies that have investigated the existence of disparities in CV events and mortality among individuals having NGT without MetS (NGT_non_MetS), those having IGT_non_MetS, and those having IGT with MetS (IGT_MetS) are limited.

To address this important knowledge gap, our study aimed to examine whether disparities exist in CV events and mortality among the following three groups: NGT_non_MetS, IGT_non_MetS, and IGT_MetS. This is the first study, to our knowledge, to investigate the disparities in both CV events and mortality among individuals with IGT_non_MetS, IGT_MetS, and NGT_non_MetS, providing valuable insights for precision prevention strategies targeting IGT.

## 2 METHODS

### 2.1 Study population

The Da Qing IGT and Diabetes Study design, methods, and population have been previously reported.^14^, ^15^ Residents of Da Qing, China, aged 25–74 years, were screened for diabetes in 1986. In total, 3956 residents were tested using a 75‐g oral glucose tolerance test (OGTT). Based on the World Health Organization (WHO) criteria of 1985, 576 adults (312 men and 264 women) were identified as having IGT. Additionally, 519 age‐ and sex‐matched participants with NGT were included as controls. After excluding 25 participants with missing baseline information in the NGT group, 36 participants with missing baseline information in the IGT group, and 23 NGT participants with MetS, 1011 participants were included in this study.

This study was approved by the institutional review boards of the WHO and China–Japan Friendship Hospital, and all surviving participants and proxies for the deceased participants provided written informed consent.

### 2.2 MetS diagnostic criteria and study groups

MetS was defined according to the American Association of Clinical Endocrinology criteria with modifications (any three of the five following criteria)^16^, ^17^: (1) hyperglycemia (type 2 DM [T2DM], impaired fasting glucose level between 6.1 and 7.0 mmol/L, or 2‐h postprandial plasma glucose level between 7.8 and 11.1 mmol/L); (2) hypertension (>130/85 mm Hg); (3) reduced high‐density lipoprotein cholesterol levels (<40 mg/dL for males and <50 mg/dL for females); (4) elevated triglyceride level (≥1.69 mmol/L); and (5) obesity (body mass index ≥30 kg/m^2^).

The participants were stratified based on their MetS status at baseline to evaluate its influence on CV events and mortality. The participants were divided into three subgroups: (1) NGT_non_MetS (471 participants); (2) IGT_non_MetS (294 participants); and (3) IGT_MetS (264 participants).

### 2.3 Data collection and outcomes

Data were collected through personal interviews and clinical examinations of living participants in 2016. Those who were unable to attend the hospital examination because of ill health or were living outside Da Qing city were interviewed by telephone and examined at home or in local hospitals. For the deceased participants, standardized questionnaires were used to interview proxy informants, such as a living spouse, sibling, or child, and the data were subsequently verified using medical records, death certificates, or both. The two primary outcomes of interest were CV events and CV mortality. CV events were defined as nonfatal or fatal myocardial infarction (MI) or sudden death, hospital admission for heart failure, and nonfatal or fatal stroke. CV mortality was defined as deaths due to MI, sudden death, heart failure, or stroke. Secondary outcomes were all‐cause mortality; MI events or MI mortality, defined as nonfatal or fatal MI; and stroke events or stroke mortality, defined as nonfatal or fatal stroke.

### 2.4 Statistical analyses

Participant characteristics are presented as mean (standard deviations) for quantitative variables and as percentages for categorical variables. The analysis of variance and chi‐squared tests were separately used for normally distributed continuous and categorical variables, respectively. Incidence rate was defined as the number of events divided by the number of person‐years. Person‐years were defined as the duration from inclusion in this study (January 1, 1986) to the date of events, death, or December 31, 2016, whichever occurred first.

Cox proportional hazards (PH) regression was used to investigate the relationship among the groups (NGT_non_MetS, IGT_non_MetS, IGT_MetS) and outcomes. Kaplan–Meier survival curves were generated to determine the time‐to‐event survival for each outcome, and log‐rank tests were used to compare differences among groups.

The PH assumption for subgroups and clinical outcomes was tested using Schoenfeld residuals, and the results met the PH assumption.^18^ In addition to providing the hazard ratio (HR) in a fully adjusted model, we adjusted the covariates stepwise. Model 1 was an unadjusted model, and model 2 was adjusted for sex, age, and smoking status.

To further explore whether there were differences in all‐cause mortality, MI events or MI mortality, and stroke events or stroke mortality, we examined the risk among the three groups. Data management and processing were performed using R software version 4.1.0. Differences were considered significant when the two‐sided test *p* value was <0.05.

## 3 RESULTS

### 3.1 Baseline characteristics

This study included 471, 294, and 246 participants in the NGT_non_MetS, IGT_non_MetS, and IGT_MetS groups, respectively, for the follow‐up analysis. The mean age of the participants was 44.57 (standard deviation, 9.07) years. In total, 55.39% of the participants were male (560 of 1011 participants). The baseline characteristics of each group are shown in Table 1. Notably, all baseline characteristics in the IGT_MetS group were significantly different from those in the NGT_non_MetS group. Conversely, there were no significant differences in baseline characteristics between the NGT_non_MetS and IGT_non_MetS groups, except for fasting plasma glucose level, 1‐h plasma glucose concentration, 2‐h plasma glucose concentration, and body mass index (BMI).

**TABLE 1:** Baseline characteristic of the NGT_non_MetS, IGT_non_MetS, and IGT_MetS groups.

Variables	NGT_non_MetS	IGT_non_MetS	IGT_MetS	*p* value^a^	*p* value^b^
*N*	471	294	246		
Male sex (%)	262 (55.63)	183 (62.24)	115 (46.75)	0.084	0.029
Age (years)	43.84 (9.01)	44.18 (8.76)	46.42 (9.34)	0.6	<0.001
BMI (kg/m^2^)	23.40 (3.07)	24.29 (3.47)	27.81 (3.62)	<0.001	<0.001
Current smoker (%)	216 (45.86)	136 (46.26)	83 (33.74)	0.97	0.002
Fasting plasma glucose (mmol/L)	4.76 (0.67)	5.51 (0.81)	5.69 (0.79)	<0.001	<0.001
1‐h PG (mmol/L)	6.68 (1.43)	11.25 (2.24)	11.25 (2.28)	<0.001	<0.001
2‐h PG (mmol/L)	5.00 (1.18)	8.94 (0.86)	9.07 (0.91)	<0.001	<0.001
Systolic blood pressure (mm Hg)	121.32 (20.63)	122.28 (20.72)	144.83 (23.57)	0.53	<0.001
Diastolic blood pressure (mm Hg)	81.25 (13.67)	81.23 (13.37)	94.64 (12.18)	0.99	<0.001
Total cholesterol (mmol/L)	4.76 (1.13)	4.95 (1.27)	5.21 (1.35)	0.055	<0.001
Triglycerides (mmol/L)	1.29 (0.99)	1.44 (1.42)	2.56 (1.96)	0.15	<0.001
High‐density lipoprotein cholesterol (mg/dL)	53.86 (11.48)	54.20 (11.42)	46.33 (10.81)	0.69	<0.001

*Note*: Data are presented as means (standard deviations) for continuous variables and *N* (%) for categorical variables. *p* values comparing means were based on analysis of variance for continuous variables and the chi‐squared test for categorical variables.

Abbreviations: 1‐h PG, venous plasma glucose concentration 1 h after 75‐g oral glucose load; 2‐h PG, venous plasma glucose concentration 2 h after 75‐g oral glucose load; BMI, body mass index; IGT_MetS, impaired glucose tolerance with metabolic syndrome; IGT_non_MetS, impaired glucose tolerance without metabolic syndrome; MI, myocardial infarction; NGT_non_MetS, normal glucose tolerance without metabolic syndrome.

^a^ NGT_non_MetS versus IGT_non_MetS.

^b^ NGT_non_MetS versus IGT_MetS.

### 3.2 The risk of CV events and mortality in the three groups

The 30‐year follow‐up data revealed the number of events and incidence rates in the NGT_non_MetS, IGT_non_MetS, and IGT_MetS groups (Table 2). An evident increasing trend was observed in the incidence rates of CV mortality from the NGT_non_MetS to the IGT_non_MetS and IGT_MetS groups, with incidence rates of 4.5, 7.66, and 12.23 per 1000 person‐years, respectively. A similar pattern was observed in CV events, with incidence rates of 15.34, 20.56, and 27.99 per 1000 person‐years, respectively.

**TABLE 2:** Incidences and hazard ratios for CV mortality and events in NGT_non_MetS, IGT_non_MetS, and IGT_MetS.

Outcomes	NGT_non_MetS (*N* = 471)	IGT_non_MetS (*N* = 294)	IGT_MetS (*N* = 246)
**CV mortality**			
**Cases/person‐years**	57/12 668	55/7176	74/6051
**Incidence/1000 person‐years (95% CI)**	4.5 (3.33–5.67)	7.66 (5.64–9.69)	12.23 (9.44–15.02)
Models, HR (95% CI)			
Unadjusted	Reference	1.78 (1.23–2.58)	2.88 (2.04–4.07)
Adjusted for age, sex, and smoking status	Reference	2 (1.38–2.91)	2.96 (2.09–4.19)
**CV events**			
Cases/person‐years	173/11 276	129/6275	141/5037
Incidence/1000 person‐years (95% CI)	15.34 (13.06–17.63)	20.56 (17.01–24.11)	27.99 (23.37–32.61)
Models, HR (95% CI)			
Unadjusted	Reference	1.39 (1.11–1.75)	1.96 (1.57–2.45)
Adjusted for age, sex, and smoking status	Reference	1.49 (1.19–1.88)	1.97 (1.58–2.47)

*Note*: Bold indicates the outcomes.

Abbreviations: CI, confidence interval; CV, cardiovascular; HR, hazard ratio; IGT_MetS, impaired glucose tolerance with metabolic syndrome; IGT_non_MetS, impaired glucose tolerance without metabolic syndrome; MI, myocardial infarction; NGT_non_MetS, normal glucose tolerance without metabolic syndrome.

**FIGURE 1:**
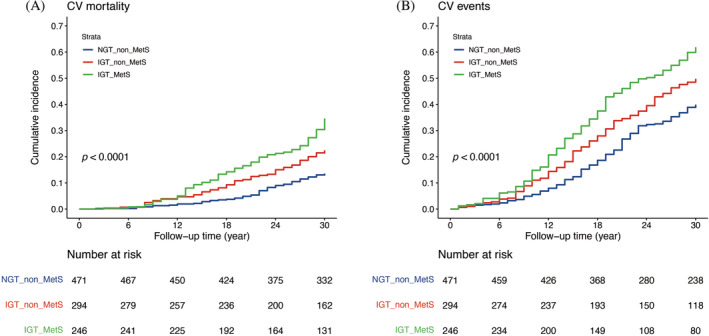
Kaplan–Meier plot of 30‐year cumulative incidence of cardiovascular (CV) mortality and events of the normal glucose tolerance without metabolic syndrome (NGT_non_MetS), impaired glucose tolerance without metabolic syndrome (IGT_non_MetS), and impaired glucose tolerance with metabolic syndrome (IGT_MetS) groups. (A) Cumulative incidence of CV mortality. (B) Cumulative incidence of CV events.

The cumulative incidences of outcomes among the NGT_non_MetS, IGT_non_MetS, and IGT_MetS groups are shown in Figure 1. As illustrated in Figure 1A, an increasing cumulative incidence trend was noted from the NGT_non_MetS group to the IGT_non_MetS group and finally to the IGT_MetS group in terms of CV mortality. A similar increasing cumulative incidence trend was observed for CV events in Figure 1B.

To ascertain whether the effect of cumulative incidence on mortality or events in the different groups was primarily attributable to their own characteristics or could be influenced by other risk factors, multivariate models were used to adjust for additional risk factors. The HRs for mortality and events are presented in Table 2. After adjusting for sex, age, and smoking status, the HRs for CV mortality in the IGT_non_MetS and IGT_MetS groups were 2 (95% confidence interval [CI], 1.38–2.91) and 2.96 (95% CI, 2.09–4.19), respectively. For CV events, after similar adjustments, the HRs for the IGT_non_MetS and IGT_MetS groups were 1.49 (95% CI, 1.19–1.88) and 1.97 (95% CI, 1.58–2.47), respectively.

### 3.3 The risk of all‐cause mortality, MI events or MI mortality, and stroke events or stroke mortality in the three groups

**FIGURE 2:**
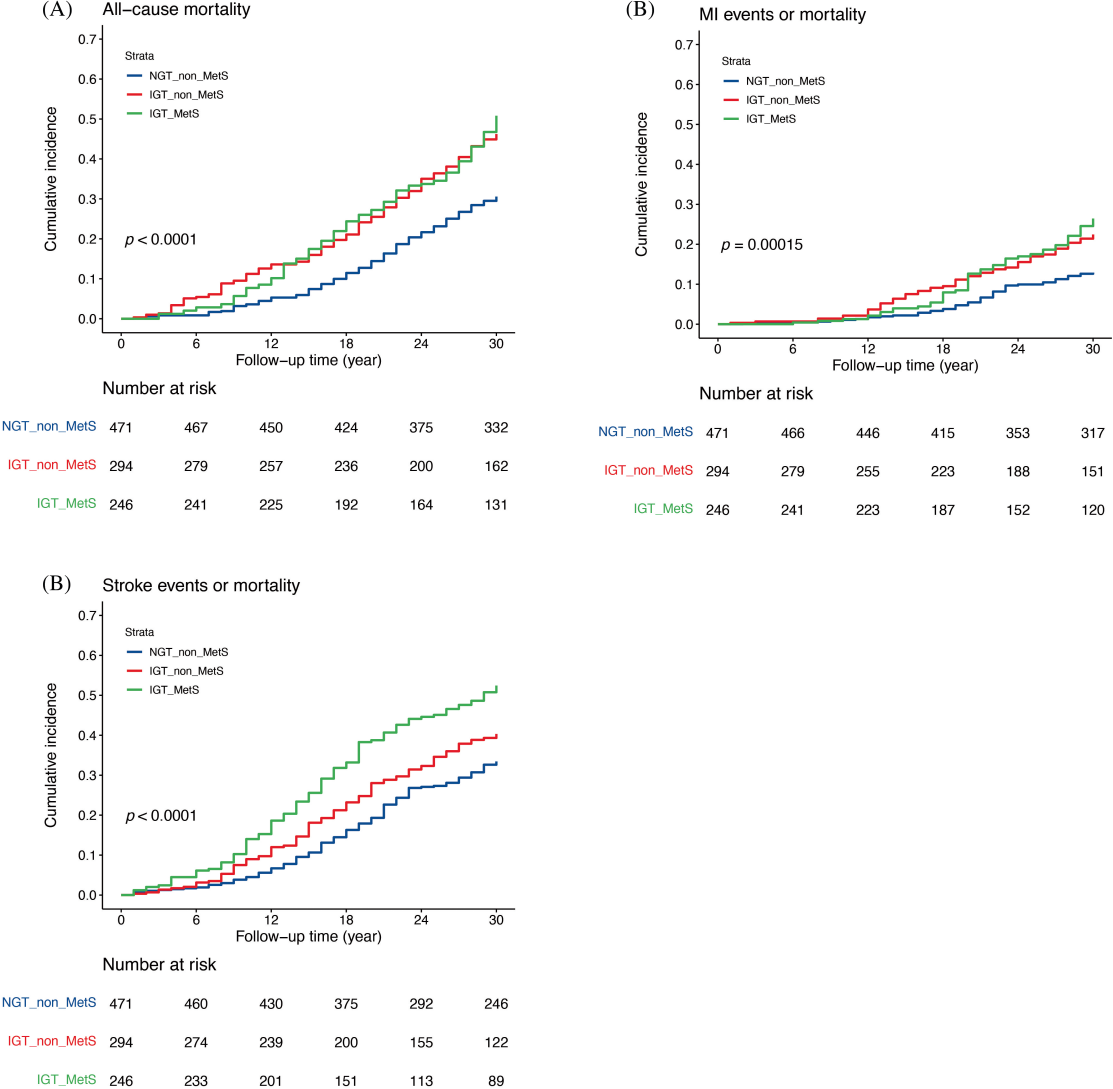
Kaplan–Meier plot of 30‐year cumulative incidence of all‐cause mortality, myocardial infarction (MI) events or MI mortality, and stroke events or stroke mortality of the normal glucose tolerance without metabolic syndrome (NGT_non_MetS), impaired glucose tolerance without metabolic syndrome (IGT_non_MetS), and impaired glucose tolerance with metabolic syndrome (IGT_MetS) groups. (A) Cumulative incidence of all‐cause mortality. (B) Cumulative incidence of MI events or MI mortality. (C) Cumulative incidence of stroke events or stroke mortality.

The results shown in Table 2 indicate significant differences in CV mortality and events among the three groups. To further explore the existence of a difference in all‐cause mortality, MI events or MI mortality, and stroke events or stroke mortality, we compared these outcomes among the three groups (Table 3). After adjusting for age, sex, and smoking status, the HRs for all‐cause mortality in the IGT_non_MetS and IGT_MetS groups were 1.98 (95% CI, 1.56–2.5) and 1.96 (95% CI, 1.54–2.5), respectively. Similar results were observed for MI events or MI mortality and stroke events or stroke mortality. The cumulative incidences of all‐cause mortality, MI events or MI mortality, and stroke events or stroke mortality are shown in Figure 2. An increasing trend was observed in the incidence rates of all‐cause mortality, MI events or MI mortality, and stroke events or stroke mortality in the IGT_non_MetS and IGT_MetS groups compared with the NGT_non_MetS group.

**TABLE 3:** Incidences and hazard ratios for all‐cause mortality, MI events or MI mortality, and stroke events or stroke mortality in the NGT_non_MetS, IGT_non_MetS, and IGT_MetS groups.

Outcomes	NGT_non_MetS (*N* = 471)	IGT_non_MetS (*N* = 294)	IGT_MetS (*N* = 246)
**All‐cause mortality**			
Cases/person‐years	144/12 668	136/7176	125/6051
Incidence/1000 person‐years (95% CI)	11.37 (9.51–13.22)	18.95 (15.77–22.14)	20.66 (17.04–24.28)
Models, HR (95% CI)			
Unadjusted	Reference	1.74 (1.37–2.2)	1.91 (1.5–2.43)
Adjusted for age, sex, and smoking status	Reference	1.98 (1.56–2.5)	1.96 (1.54–2.5)
**MI events or MI mortality**			
Cases/person‐years	53/12 398	53/6968	50/5884
Incidence/1000 person‐years (95% CI)	4.27 (3.12–5.43)	7.61 (5.56–9.65)	8.5 (6.14–10.85)
Models, HR (95% CI)			
Unadjusted	Reference	1.87 (1.28–2.74)	2.12 (1.44–3.13)
Adjusted for age, sex, and smoking status	Reference	2.03 (1.39–2.98)	2.16 (1.46–3.19)
**Stroke events or stroke mortality**			
Cases/person‐years	143/11 450	101/6384	117/5124
Incidence/1000 person‐years (95% CI)	12.49 (10.44–14.54)	15.82 (12.74–18.91)	22.83 (18.7–26.97)
Models, HR (95% CI)			
Unadjusted	Reference	1.31 (1.01–1.69)	1.94 (1.52–2.47)
Adjusted for age, sex, and smoking status	Reference	1.39 (1.08–1.8)	1.97 (1.54–2.52)

*Note*: Bold indicates the outcomes.

Abbreviations: CI, confidence interval; HR, hazard ratio; IGT_MetS, impaired glucose tolerance with metabolic syndrome; IGT_non_MetS, impaired glucose tolerance without metabolic syndrome; MI, myocardial infarction; NGT_non_MetS, normal glucose tolerance without metabolic syndrome.

## 4 DISCUSSION

We used the 30‐year follow‐up data to investigate CV mortality and events among the following three groups of participants: NGT_non_MetS, IGT_non_MetS, and IGT_MetS. Our study revealed significantly higher CV mortality and events in the IGT_non_MetS and IGT_MetS groups compared with the NGT_non_MetS group. This suggests that active intervention is required. In our sensitivity analysis, we found that the IGT_non_MetS and IGT_MetS groups had higher risks of all‐cause mortality, MI events or MI mortality, and stroke events or stroke mortality, compared with the NGT_non_MetS group. This finding suggested that the IGT_non_MetS and IGT_MetS groups have increased all‐cause mortality, MI events or MI mortality, and stroke events or stroke mortality.

Our study demonstrated that the IGT_non_MetS and IGT_MetS groups had an increased risk of CV mortality and events. This is consistent with previous research showing that prediabetes was associated with both CV events and all‐cause mortality.^19^, ^20^ We observed that the baseline characteristics of the NGT_non_MetS and IGT_non_MetS groups differed only in blood glucose level and BMI; in the case of only high blood glucose level and BMI, the risk of CV mortality and events in the IGT_non_MetS group increased. Moreover, we found that the IGT_MetS group had a significantly higher HR for CV mortality compared with the IGT_non_MetS group (data not shown). This result implies that MetS partially mediates CV mortality in individuals with IGT and supports the hypothesis of a previous study.^13^ These results demonstrated that high glucose levels and BMI in the IGT_non_MetS group increased the risk of CV mortality and events, whereas IGT_MetS group was associated with a higher risk of CV mortality and events.

Although MetS mediates CV mortality risk, differences were observed in the risk between the NGT_non_MetS and IGT_non_MetS groups. Additionally, previous studies have suggested that the association between prediabetes and CV events may only be present in IGT with hypertension compared with NGT.^21^, ^22^, ^23^ Our results revealed that IGT_non_MetS with elevated blood glucose levels and BMI compared with NGT_non_MetS still contributes to increased CV mortality and event risks. This difference may be due to differences in the follow‐up time.

Although the initiation of interventions and frequent follow‐up are recommended, the presence of IGT is considered a risk factor rather than a clinical entity in its own right.^24^ Despite several studies demonstrating that lifestyle/behavioral intervention with an individualized reduced‐calorie meal plan is highly effective in preventing or delaying T2DM and improving other cardiometabolic markers,^25^, ^26^, ^27^ the prevalence of diabetes continues to increase. This suggests that individuals may not pay sufficient attention to their IGT status.^28^ Our findings further underscore the significantly increased risk of CV mortality and events in the IGT_non_MetS and IGT_MetS population. Furthermore, our study revealed a noteworthy finding that the IGT_MetS group had a higher risk of CV mortality than that of the IGT_non_MetS group. These findings provide evidence for the need for precise and targeted interventions in patients with IGT. Consequently, tailoring interventions according to whether IGT is accompanied by MetS can potentially maximize cost‐effectiveness, especially within the constraints of limited medical resources.

The key strength of this study is that the 30‐year follow‐up period yielded an adequate number of CV deaths. Moreover, we used a standardized 75‐g OGTT to identify participants with IGT and NGT based on residents. A key limitation of this study is the use of a single definition of MetS. The absence of waist circumference data for the study population limited the use of other MetS criteria. Furthermore, systematic evaluation of the clinical outcomes of the participants at regular intervals was not feasible. Lastly, the transition to MetS indeed impacts CV events and mortality. The primary objective of this study was to explore how different baseline states—NGT_non_MetS, IGT_non_MetS, and IGT_MetS—impact CV events and mortality. Concurrently, there was a strong correlation between the baseline state and the transition to MetS. Focusing solely on the baseline state without considering the progression to MetS may represent an inherent limitation of this type of study.

Our study demonstrated that the IGT_MetS group has a significantly higher risk of CV mortality and events, compared with the NGT_non_MetS group. Although MetS mediates an important part of the risk of CV death and events, patients with IGT_non_MetS also have a higher risk of CV death and events, compared with patients with NGT_non_MetS. These findings indicate that IGT_non_MetS and IGT_MetS have a significant risk of CV events and death and that active intervention is required.

## AUTHOR CONTRIBUTIONS

*Conception or design*: F.C., Y.H., G.L., and B.Z. *Acquisition, analysis, or interpretation of data*: F.C., Y.H., J.W., Q.G., Y.C., Y.A., S.H., and L.Y. *Drafting the work or revising*: F.C. and Y.H. *Final approval of the manuscript*: B.Z.

## FUNDING INFORMATION

This study was supported by National High Level Hospital Clinical Research Funding (2022‐NHLHCRF‐YS‐01).

## CONFLICT OF INTEREST STATEMENT

The authors declare no conflicts of interest.
